# A Prenatal Multiple Micronutrient Supplement Produces Higher Maternal Vitamin B-12 Concentrations and Similar Folate, Ferritin, and Zinc Concentrations as the Standard 60-mg Iron Plus 400-μg Folic Acid Supplement in Rural Bangladeshi Women^1^,^2^

**DOI:** 10.3945/jn.116.235994

**Published:** 2016-10-19

**Authors:** Shirin Ziaei, Anisur Rahman, Rubhana Raqib, Bo Lönnerdal, Eva-Charlotte Ekström

**Affiliations:** 3 Department of Women’s and Children’s Health, Uppsala University, Uppsala, Sweden;; 4 International Center for Diarrheal Disease Research, Bangladesh (ICDDR,B), Dhaka, Bangladesh; and; 5 Department of Nutrition, University of California, Davis, Davis, CA

**Keywords:** efficacy, effectiveness, prenatal micronutrient supplementation, food supplementation, maternal micronutrient status, folate, ferritin, vitamin B-12, zinc, Bangladesh

## Abstract

**Background:** The effects of prenatal food and micronutrient supplementation on maternal micronutrient status are not well known.

**Objective:** We compared the efficacy and effectiveness of 3 different micronutrient supplements on maternal micronutrient status when combined with food supplementation.

**Methods:** In the MINIMat (Maternal and Infant Nutrition Intervention, Matlab) trial in Bangladesh, 4436 pregnant women were randomly assigned to daily intake of 3 types of micronutrient capsules: 30 mg Fe and 400 μg folic acid (Fe30F), 60 mg Fe and 400 μg folic acid (Fe60F), or multiple micronutrient supplements (MMNs) combined with early (week 9 of pregnancy) or usual (week 20 of pregnancy) food supplementation in a 2 by 3 factorial design. Plasma concentrations of vitamin B-12, folate, ferritin, and zinc were analyzed before the start of micronutrient supplementation (week 14) and at week 30 of pregnancy in 641 randomly selected women. An electronic monitoring device was used to measure the number of capsules taken. The effectiveness of food and micronutrient regimens as well as efficacy per capsule in maternal micronutrient status were analyzed by ANOVA and general linear models.

**Results:** At week 30 of pregnancy, women in the MMN group had higher geometric mean concentrations of vitamin B-12 than women in the Fe60F group (119 compared with 101 pmol/L, respectively); no other differences in effectiveness of micronutrient and food regimens were observed. A dose-response relation between the number of capsules taken and concentrations of folate and ferritin was observed for all micronutrient supplements. Fe30F had lower efficacy per capsule in increasing ferritin concentrations within the first tertile of capsule intake than did Fe60F and MMNs. Because ferritin reached a plateau for all types of micronutrient supplements, there was no difference between the regimens in their effectiveness.

**Conclusion:** Compared with Fe60F, MMNs produced higher maternal vitamin B-12 and similar ferritin and folate concentrations in Bangladeshi women. The MINIMat trial was registered at isrctn.org as ISRCTN16581394.

## Introduction

Recommendations for iron and folic acid supplementation during pregnancy have been in place since 1968, albeit with variations in doses (1). Despite these longstanding recommendations, iron deficiency during pregnancy remains globally prevalent and is reported to be responsible for >115,000 maternal deaths annually and 0.4% of total disability-adjusted life-years (2, 3). In disadvantaged settings, regardless of iron status, the coexistence of other micronutrient deficiencies is common during pregnancy (4) and may limit the effectiveness of iron and folic acid supplementation (5) as well as result in both short- and long-term health consequences for the offspring (6–8). To reduce the risk of multiple micronutrient deficiencies and to protect both mothers and children, a prenatal multiple micronutrient supplement (MMN)^6^ has been developed and promoted by UNICEF, the WHO, and the United Nations University (9).

Despite the lower daily dose of iron in MMNs (30 mg), regimens that use MMNs have shown effects similar to standard 60-mg Fe and 400-μg folic acid regimens on maternal anemia and on plasma ferritin (4, 10, 11). It is possible that additional micronutrients present in MMNs may improve iron absorption and utilization. Another possible explanation is that the lower dose of iron in MMNs might be sufficient to reach a maximum hematologic effect over the duration of the supplementation regimen. Previous studies by our group (12, 13) suggest that a considerably lower amount of iron than is currently recommended is required to reach a plateau in hematologic response. Understanding the patterns of dose-response between intakes of different types of micronutrient supplements and maternal micronutrient status would enable the estimation of the lowest amounts of micronutrients required to achieve optimal status.

The MINIMat (Maternal and Infant Nutrition Intervention, Matlab) trial in pregnant women in Matlab, Bangladesh, was designed to evaluate the effects of different types of prenatal micronutrient regimens in combination with the timing of food supplementation. Although previous reports from this trial showed a high prevalence of anemia, zinc, and vitamin B-12 deficiencies in early pregnancy among the women in the trial (14), there was no differential effect of using the MMN regimen on women’s hemoglobin concentration compared with standard iron–folic acid supplementation regimens (11). In this article, our aims were to compare the effectiveness of different prenatal food and micronutrient regimens as well as the efficacy per micronutrient capsule in women’s vitamin B-12, folate, ferritin, and zinc status.

## Methods

### 

This study was part of a larger study, the MINIMat trial (isrctn.org, ISRCTN16581394). The design and procedures of the MINIMat trial have been described in detail previously (11). In brief, the study area was Matlab, a rural subdistrict in Bangladesh, which is a field site of the International Center for Diarrheal Diseases Research, Bangladesh (ICDDR,B). Since 1966, a Health and Demographic Surveillance System has been in place, covering a population of ∼220,000 individuals, and records their demographic and selected health information on a monthly basis. During November 2001–October 2003, all women who were identified and confirmed pregnant were invited to attend the MINIMat trial. The women were included in the trial if they were had been pregnant <14 wk, had no severe illnesses, and gave written consent.

Over a 2-y period, 4436 pregnant women were enrolled in the MINIMat trial and allocated to 2 types of food supplementation and 3 types of micronutrient supplementation regimes in a randomized 2 by 3 factorial design. The food supplement was provided by the ongoing government-supported national program in Matlab that provided food supplements to all pregnant women attending the community nutrition centers. The locally produced protein-energy supplement was provided 6 d/wk regardless of the women’s BMI. Each package of food supplement contained 80 g roasted rice powder, 40 g roasted pulse powder, 20 g molasses, and 12 mL (6 g) soybean oil, which provided 608 kcal of energy and 18 g vegetable protein (contributing 12% of energy). Each woman was randomly assigned to be individually invited to start food supplementation as soon as her pregnancy was detected (approximately week 9 of pregnancy; early food) or to start at a time of her own choosing (usual food), commonly at approximately week 20 of pregnancy. Across the food supplementation groups, the women were randomly assigned to receive 1 of 3 types of identical-looking micronutrient capsules from week 14 in pregnancy, as follows: *1*) 30 mg Fe and 400 μg folic acid (Fe30F), *2*) 60 mg Fe and 400 μg folic acid (Fe60F), or *3*) MMNs containing 30 mg Fe, 400 μg folic acid, 2.6 μg vitamin B-12, 15 mg Zn, and 1 RDA of 11 other micronutrients (MMNs) (9).

Information on socioeconomic status, education, and pregnancy history was collected by using a precoded questionnaire administered at a household visit at approximately week 8 of pregnancy. Participating women were scheduled to visit health centers at weeks 14 and 30 of pregnancy, at which time venous blood samples were collected. Plasma samples were centrifuged, separated, and stored at –70°C in freezers in Matlab until they were shipped on dry ice to the University of California, Davis, where the biomarkers were analyzed. Plasma ferritin was analyzed by RIA (Diagnostic Products). The plasma concentration of zinc was measured by atomic absorption spectrometry (15). Plasma vitamin B-12 and folate were determined simultaneously by using an RIA (SimulTRAC-SNB; MP Biomedicals). To analyze the secondary objective of effects of supplementation on maternal micronutrient status, we used a subset of the women who were enrolled in the trial during the calendar year of 2002 (1 January to 31 December; *n* = 2119) and who were assigned to become a cohort for the assessment of biomarkers. From this cohort, 1000 of the enrolled women were randomly selected for the assessment of micronutrients. The basis was to allow up to 25% attrition after enrollment and still enable a biologically important difference (arbitrarily set to a *z* score of 0.25) to be detected between the 3 micronutrient supplementation groups in a 2-tailed test with 95% probability and 80% power. Results from 1 set of laboratory analysis comprising 130 randomly selected women were lost due to technical problems, resulting in a total of 871 randomly selected enrolled women. After attrition, 641 biological samples were available for analyses, which enabled a *z* score difference of 0.27 to be detected between the micronutrient groups.

The number of capsules taken was estimated by the assessment of capsule bottle openings from the time of distribution to week 30 with the use of an electronic drug-monitoring device (eDEM; Aprex). The device (installed in the cap) recorded the date and time of each bottle opening. Bottles contained 35 capsules and were replaced monthly by home interviewers. After collecting the capsule bottles from the pregnant women, data were downloaded and transferred into a computer program. To avoid the potential overestimation of capsule intake that may have occurred due to multiple openings per day without taking any capsule, openings that occurred on the day of distribution of the bottle were excluded from analyses. In addition, >3 openings during a single day were recoded and analyzed as an intake of 3 capsules. The number of food supplements consumed was estimated by monthly self-reported recall.

The study followed the principles of the Declaration of Helsinki. The women were enrolled after giving their written informed consent, and they were told that they could withdraw from the study at any time. Confidentiality of information was followed throughout the whole process. The study was approved by the ethical review committee at the ICDDR,B. There were no connections to Uppsala University at the time of the trial.

Enrollment characteristics of the participating women and those who were excluded from the study were compared by using Student’s *t* test or ANOVA for continuous variables and chi-square test for categorical variables. The normality of data distribution was checked by visual examination of histogram and Q-Q plots; plasma concentrations of micronutrients were highly skewed and thus were transformed to natural logarithms before statistical analyses and reported as geometric means for descriptive results and log_e_ values for differences in means.

The comparison of effectiveness between supplementation regimens was done by intent-to-treat analysis, and concentrations of micronutrients at week 30 of pregnancy were compared by using general linear models. The currently recommended Fe60F and usual invitation to food supplements were considered to be the reference groups. We checked for potential interaction terms between food and micronutrient regimens by adding interactions in the models.

In the next step of analyses, patterns of efficacy (i.e., dose-response per capsule) were evaluated within each micronutrient supplement type. Locally weighted scatterplot smoothing (Lowess) curves were used to visualize relations between the number of capsules taken by week 30 of pregnancy and plasma concentrations of micronutrients, and linear regression models were used to evaluate whether a dose-response relation existed over the full range of capsule intake. Because Lowess curves suggested nonlinear relations for some of the micronutrients, we also used general linear models to compare mean concentrations of micronutrients between tertiles of capsule intake within each supplementation type. Furthermore, because dose-response for ferritin indicated a plateau, the lowest tertile of capsule intake (where a dose-response was observed) was selected and dose-response per capsule between different micronutrient types was modeled as a function of number of capsules taken, supplementation type, and interaction between number of capsules and supplementation type. All of the regression models were adjusted for the following factors: maternal formal years of education (none, 1–5 y, or ≥6 y), socioeconomic status (quintiles), food supplementation, micronutrient concentration at week 14, and duration of supplementation period. Maternal gestational age was not included in the final models because it did not alter the model variables. Significance was defined as *P* < 0.05, with interactions considered significant at *P* < 0.10 (16). Statistical analyses were conducted by using the Statistical Package for the Social Sciences (IBM SPSS statistics version 20).

## Results

### 

**FIGURE 1:**
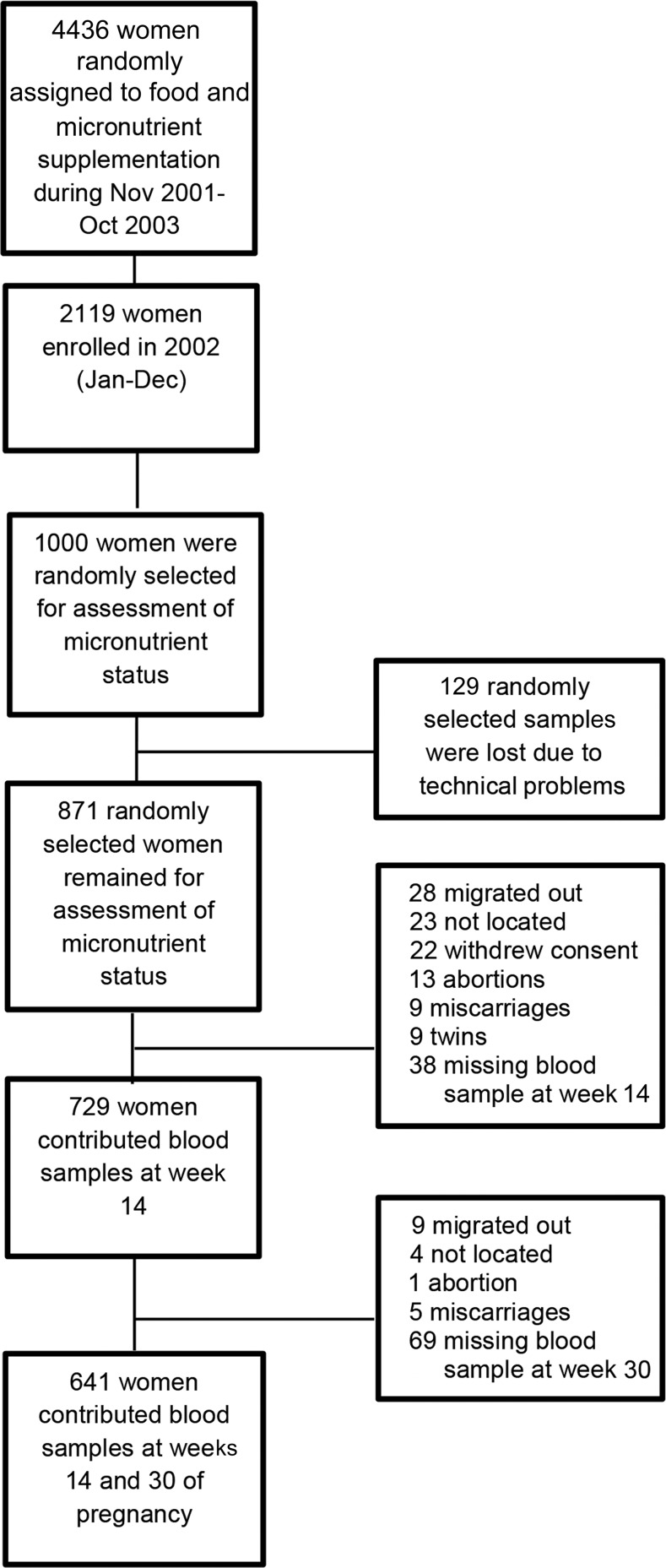
Flowchart of participating women.

Of the 871 randomly selected enrolled pregnant women, 641 singleton women contributed blood samples at week 14 and at follow-up at week 30 (**Figure 1**). The main reasons for loss to follow-up were outmigration (*n* = 35), not being able to locate the women (*n* = 27), and refusal to participate (*n* = 22). Women who did not provide a blood sample at week 14 (*n* = 38) or week 30 (*n* = 69) were excluded from the analysis. Enrollment characteristics of the final sample of women were not significantly different from the women who were not included in the analyses. Participating women were ∼27 y of age and, on average, 150 cm in height and weighed 45 kg. One-quarter had a BMI (in kg/m^2^) <18.5 and one-third of the women had no formal education. Mean concentrations of the micronutrients at week 14 did not differ between the supplementation regimens (**Table 1**).

**TABLE 1:** Enrollment characteristics and adherence to micronutrient supplementation by supplementation type in a selected sample of rural Bangladeshi pregnant women participating in the MINIMat trial^1^

	Early invitation to food supplementation			Usual invitation to food supplementation			
Maternal characteristics	Fe30F (*n* = 98)	Fe60F (*n* = 104)	MMN (*n* = 111)	Fe30F (*n* = 115)	Fe60F (*n* = 112)	MMN (*n* = 101)	*P*^2^
Age, y	26.0 ± 6.21^3^	27.2 ± 6.28	27.0 ± 6.22	26.9 ± 5.88	26.6 ± 6.02	26.1 ± 5.41	0.62
Initial body weight, kg	45.9 ± 6.40	46.2 ± 6.18	46.0 ± 7.39	44.9 ± 6.73	45.5 ± 6.79	47.7 ± 6.90	0.06
Height, cm	149 ± 5.51	151 ± 5.07	150 ± 5.55	149 ± 5.21	149 ± 5.66	150 ± 5.51	0.09
Enrollment BMI, kg/m^2^	20.2 ± 2.36	20.2 ± 2.38	20.3 ± 2.87	20.1 ± 2.61	20.3 ± 2.61	21.0 ± 2.65	0.12
Years of schooling, *n* (%)							0.97
0	31 (31.6)	32 (30.8)	38 (34.2)	38 (33.0)	34 (30.4)	34 (33.7)	
1–5	23 (23.5)	24 (23.1)	21 (18.9)	25 (21.7)	19 (17.0)	23 (22.8)	
≥6	44 (44.9)	48 (46.2)	52 (46.8)	52 (45.2)	59 (52.7)	44 (43.6)	
SES, *n* (%)							0.87
1 (poorest)	19 (19.4)	20 (19.2)	25 (22.5)	21 (18.3)	24 (21.4)	10 (9.9)	
2 (poorer)	25 (25.5)	24 (23.1)	23 (20.7)	23 (20.0)	20 (17.9)	25 (24.8)	
3 (middle)	15 (15.3)	23 (22.1)	18 (16.2)	25 (21.7)	23 (20.5)	27 (26.7)	
4 (richer)	17 (17.3)	16 (15.4)	21 (18.9)	23 (20.0)	21 (18.8)	18 (17.8)	
5 (richest)	22 (22.4)	21 (20.2)	24 (21.6)	23 (20.0)	24 (21.4)	21 (20.8)	
Capsule intake, *n*	72.2 ± 36.4	76.6 ± 33.4	77.8 ± 31.1	85.4 ± 34.6	79.7 ± 32.8	83.9 ± 31.2	0.08
Duration of supplementation, d	114 ± 10.4	113 ± 7.91	115 ± 10.6	114 ± 9.40	113 ± 10.1	113 ± 8.21	0.53

1 Fe30F, 30 mg Fe and 400 μg folic acid; Fe60F, 60 mg Fe and 400 μg folic acid; MINIMat, Maternal and Infant Nutrition Intervention, Matlab; MMN, multiple micronutrient supplement; SES, socioeconomic status.

2 Comparison between groups was made by ANOVA for continuous variables and by chi-square test for categorical variables.

3 Mean ± SD (all such values).

By design, women in the early food group consumed more packages of food supplements than did the usual food supplementation group (87 ± 42 compared with 59 ± 37, respectively). On average, women took 79 ± 34 micronutrient capsules from week 14 to follow-up at week 30 (114 ± 9 d) and the mean percentage of compliance (defined as the mean number of capsules taken/duration of supplementation in days ×100) was 70%. No significant difference was observed in mean percentage of compliance between the 3 micronutrient supplementation regimens. Women who were randomly assigned to the early food supplementation regimen took 7 micronutrient capsules less, on average, than did those with the usual food supplementation regimen (*P* = 0.03). There was no interaction between the food regimen and micronutrient supplementation that affected the number of capsules taken (*P* = 0.42).

#### Comparison between food and micronutrient supplementation regimens.

As a first step, we compared maternal concentrations of micronutrients at week 30 of pregnancy among supplementation regimens. No significant differences in maternal plasma concentrations of micronutrients at week 14 or week 30 were observed when comparing early and usual invitation to food supplementation (**Table 2**). The mean plasma vitamin B-12 concentration was significantly higher among women in the MMN regimen at week 30 of pregnancy, but no other significant differences were observed in plasma concentrations of folate, ferritin, and zinc between the different micronutrient supplementation regimens (Table 2). There were no food and micronutrient supplement interactions in mean concentration of micronutrients at week 30 of pregnancy, indicating that the effect of micronutrient supplementation did not differ by food regimen.

**TABLE 2:** Plasma concentrations of micronutrients at weeks 14 and 30 of pregnancy by supplementation regimen in a selected sample of rural Bangladeshi women participating in the MINIMat trial^1^

	Maternal micronutrient supplementation				Maternal food supplementation		
	Fe30F (*n* = 213)	Fe60F (*n* = 216)	MMN (*n* = 212)	*P*^2^	Early invitation (*n* = 313)	Usual invitation (*n* = 328)	*P*^2^
Vitamin B-12							
14 wk pregnancy,^3^ pmol/L	151 (141, 164)	147 (133, 161)	150 (138, 162)	0.84	153 (144, 164)	145 (136, 156)	0.28
Mean difference^4^	Reference	−0.04 (−0.16, 0.08)	−0.02 (−0.14, 0.10)		0.05 (−0.04, 0.15)	Reference	
30 wk pregnancy,^3^ pmol/L	101 (94, 110)	110 (101, 120)	119 (110, 128)	0.02	112 (105, 120)	108 (100, 114)	0.36
Mean difference^4^	Reference	0.09 (−0.03, 0.20)	0.16 (0.04, 0.28)**		0.04 (−0.05, 0.14)	Reference	
Folate							
14 wk pregnancy,^3^ nmol/L	10.4 (9.78, 11.1)	10.4 (9.68, 11.1)	10.4 (9.68, 11.1)	1.00	10.7 (10.1, 11.2)	10.2 (9.68, 10.7)	0.20
Mean difference^4^	Reference	−0.00 (−0.09, 0.09)	−0.00 (−0.09, 0.09)		0.05 (−0.02, 0.12)	Reference	
30 wk pregnancy,^3^ nmol/L	20.7 (18.9, 22.7)	21.3 (19.7, 23.1)	21.8 (20.1, 23.3)	0.69	20.7 (19.3, 22.2)	21.8 (20.3, 23.1)	0.37
Mean difference^4^	Reference	0.03 (−0.08, 0.14)	0.05 (−0.06, 0.16)		−0.04 (−0.13, 0.05)	Reference	
Ferritin							
14 wk pregnancy,^3^ μg/L	31.8 (28.8, 35.2)	34.5 (31.5, 37.7)	34.5 (31.2, 37.7)	0.41	34.5 (31.8, 37.3)	32.8 (30.3, 35.12)	0.37
Mean difference^4^	Reference	0.08 (−0.05, 0.21)	0.08 (−0.06, 0.21)		0.05 (−0.06, 0.16)	Reference	
30 wk pregnancy,^3^ μg/L	16.1 (14.4, 17.8)	14.9 (13.6, 16.4)	16.1 (14.7, 17.5)	0.50	16.0 (14.4, 17.3)	15.5 (14.3, 16.8)	0.58
Mean difference^4^	Reference	−0.07 (−0.21, 0.06)	−0.00 (−0.14, 0.14)		0.03 (−0.08, 0.14)	Reference	
Zinc							
14 wk pregnancy,^3^ μmol/L	8.41 (8.08, 8.76)	8.58 (8.25, 8.94)	8.58 (8.25, 9.02)	0.75	8.50 (8.25, 8.85)	8.50 (8.25, 8.85)	0.96
Mean difference^4^	Reference	0.02 (−0.04, 0.08)	0.02 (−0.04, 0.08)		−0.00 (−0.05, 0.05)	Reference	
30 wk pregnancy,^3^ μmol/L	7.54 (7.24, 7.92)	7.61 (7.32, 8.00)	7.77 (7.54, 8.08)	0.63	7.69 (7.46, 8.00)	7.61 (7.39, 7.92)	0.69
Mean difference^4^	Reference	0.01 (−0.05, 0.07)	0.03 (−0.03, 0.09)		0.01 (−0.04, 0.06)	Reference	

1 **P < 0.01. Fe30F, 30 mg Fe and 400 μg folic acid; Fe60F, 60 mg Fe and 400 μg folic acid; MINIMat, Maternal and Infant Nutrition Intervention, Matlab; MMN, multiple micronutrient supplement.

2 Difference in mean of micronutrients between supplementation regimens, analyzed by ANOVA or *t* test.

3 Values are geometric means (95% CIs).

4 Values are log_e_ mean differences (95% CIs) with the Fe60F and usual invitation to food supplementation regimen as the references, analyzed by using general linear models.

#### Evaluation of efficacy and dose-response patterns within micronutrient supplementation types.

**FIGURE 2:**
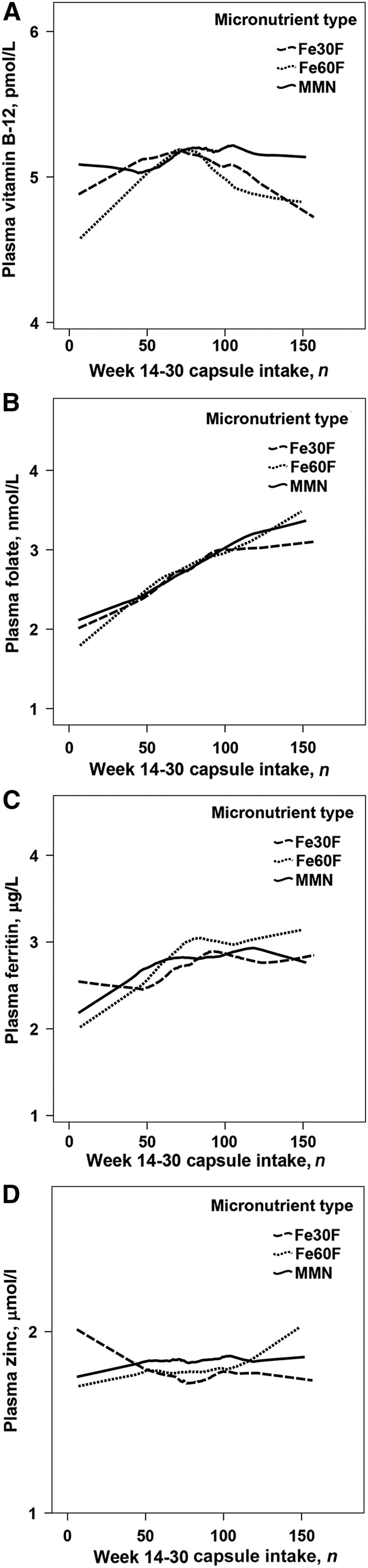
Lowess curves of associations between total numbers of capsules consumed from week 14 to 30 of pregnancy and maternal plasma concentrations of vitamin B-12 (A), folate (B), ferritin (C), and zinc (D) at week 30 of pregnancy, by micronutrient supplementation type. Plasma concentrations of micronutrients are presented in log_e_ scale. Fe30F, 30 mg Fe and 400 μg folic acid; Fe60F, 60 mg Fe and 400 μg folic acid; MMN, multiple micronutrient supplement.

As a second step, plasma concentrations of micronutrients at week 30 of pregnancy were plotted as a function of total number of capsules taken by week 30 (**Figure 2**A–D). Efficacy (i.e., dose-response per capsule) for the total number of capsules and maternal plasma concentrations of micronutrients at week 30 of pregnancy was estimated within each micronutrient supplementation type. Furthermore, mean concentrations of micronutrients were compared between tertiles of capsule intake for each micronutrient supplementation type for which the ranges of the lowest, middle, and highest tertiles of capsule intake were 6–65, 66–99, and 100–157, respectively.

#### Vitamin B-12 and dose-response.

From the graphical display, no linear dose-response relation between the number of capsules taken and maternal concentrations of plasma vitamin B-12 was observed for any of the micronutrient supplementation types (Figure 2A). This was confirmed by regression analyses when evaluating dose-response over the full range of capsule intake because there were no linear associations between the number of capsules taken and plasma concentration of vitamin B-12 for any of the micronutrient supplementation types (results not shown). Furthermore, there were no significant differences in vitamin B-12 concentration between tertiles of capsule intake within any of the micronutrient supplementation types (**Table 3**).

**TABLE 3:** Plasma vitamin B-12 concentrations at week 30 of pregnancy by tertile of capsule intake and micronutrient supplementation type in a selected sample of rural Bangladeshi women participating in the MINIMat trial^1^

Micronutrient type	Lowest tertile (6–65 capsules)	Middle tertile (66–99 capsules)	Highest tertile (100–157 capsules)
Fe30F			
* n*	60	54	65
Mean,^2^ pmol/L	103 (88, 120)	120 (87, 141)	101 (87, 119)
Unadjusted mean difference^3^	Reference	0.15 (−0.08, 0.38)	−0.01 (−0.23, 0.21)
Adjusted mean difference^4^	Reference	0.23 (−0.01, 0.47)	0.02 (−0.22, 0.25)
Fe60F			
* n*	67	65	61
Mean,^2^ pmol/L	94 (81, 109)	112 (96, 130)	98 (85, 116)
Unadjusted mean difference^3^	Reference	0.17 (−0.04, 0.39)	0.05 (−0.17, 0.26)
Adjusted mean difference^4^	Reference	0.18 (−0.04, 0.40)	−0.00 (−0.23, 0.22)
MMN			
* n*	52	69	67
Mean,^2^ pmol/L	103 (87, 119)	124 (108, 143)	128 (111, 147)
Unadjusted mean difference^3^	Reference	0.19 (−0.01, 0.40)	0.22 (0.02, 0.43)*
Adjusted mean difference^4^	Reference	0.16 (−0.05, 0.38)	0.15 (−0.07, 0.37)

1 Data were analyzed by using general linear models. *Different from the lowest tertile, *P* < 0.05. Fe30F, 30 mg Fe and 400 μg folic acid; Fe60F, 60 mg Fe and 400 μg folic acid; MINIMat, Maternal and Infant Nutrition Intervention, Matlab; MMN, multiple micronutrient supplement.

2 Values are geometric means (95% CIs).

3 Values are log_e_ mean differences (95% CIs).

4 Models adjusted for maternal formal years of education (none, 1–5 y, or ≥6 y), socioeconomic status (quintiles), food supplementation, week 14 micronutrient concentration (log_e_), and duration of supplementation.

#### Folate and dose-response.

With the use of a Lowess plot, we observed a linear dose-response relation between the number of capsules taken and plasma concentrations of folate at week 30 of pregnancy for all micronutrient supplementation types (Figure 2B). For all 3 micronutrient types, regression analyses confirmed a significant positive association between the total number of capsules taken by week 30 of pregnancy and maternal plasma folate concentration [adjusted β coefficient (β_adj_) (95% CI): 0.009 (0.007, 0.011), 0.010 (0.008, 0.013), and 0.010 (0.008, 0.012) for the Fe30F, Fe60F, and MMN groups, respectively; results not shown]. Within each micronutrient supplementation type, women in the middle and highest tertiles of capsule intake had higher mean plasma folate concentrations than did the women in the lowest tertile (**Table 4**). Among supplementation types, there was neither a difference in dose-response when evaluated over the full range of capsule intake nor when evaluating within each tertile of capsule intake (results not shown).

**TABLE 4:** Plasma folate concentrations at week 30 of pregnancy by tertile of capsule intake and micronutrient supplementation type in a selected sample of rural Bangladeshi women participating in the MINIMat trial^1^

Micronutrient type	Lowest tertile (6–65 capsules)	Middle tertile (66–99 capsules)	Highest tertile (100–157 capsules)
Fe30F			
* n*	61	55	67
Mean,^2^ nmol/L	14.2 (12.0, 16.8)	23.1 (20.7, 26.1)	28.5 (26.3, 30.9)
Unadjusted mean difference^3^	Reference	0.52 (0.33, 0.70)**	0.71 (0.53, 0.89)**
Adjusted mean difference^4^	Reference	0.51 (0.31, 0.70)**	0.70 (0.50, 0.90)**
Fe60F			
* n*	69	66	62
Mean,^2^ nmol/L	13.9 (12.3, 15.8)	24.5 (21.5, 27.9)	29.1 (25.5, 33.1)
Unadjusted mean difference^3^	Reference	0.56 (0.38, 0.75)**	0.74 (0.55, 0.92)**
Adjusted mean difference^4^	Reference	0.55 (0.36, 0.74)**	0.68 (0.49, 0.87)**
MMN			
* n*	53	69	68
Mean,^2^ nmol/L	13.7 (12.2, 15.5)	22.7 (20.5, 25.0)	30.9 (27.7, 34.1)
Unadjusted mean difference^3^	Reference	0.50 (0.34, 0.65)**	0.81 (0.65, 0.96)**
Adjusted mean difference^4^	Reference	0.50 (0.34, 0.65)**	0.78 (0.61, 0.94)**

1 Data were analyzed by using general linear models. **Different from the lowest tertile, *P* < 0.01. Fe30F, 30 mg Fe and 400 μg folic acid; Fe60F, 60 mg Fe and 400 μg folic acid; MINIMat, Maternal and Infant Nutrition Intervention, Matlab; MMN, multiple micronutrient supplement.

2 Values are geometric means (95% CIs).

3 Values are log_e_ mean differences (95% CIs).

4 Models adjusted for maternal formal years of education (none, 1–5 y, or ≥6 y), socioeconomic status (quintiles), food supplementation, week 14 micronutrient concentration (log_e_), and duration of supplementation in the analyses.

#### Ferritin and dose-response.

Regression analysis showed a dose-response relation between the number of capsules taken and maternal plasma ferritin at week 30 of pregnancy for all 3 micronutrient supplementation types [β_adj_ (95% CI): 0.003 (0.001, 0.006), 0.008 (0.005, 0.011), and 0.005 (0.002, 0.007) for the Fe30F, Fe60F, and MMN groups, respectively; results not shown]. Graphical display (Figure 2C) indicated that plasma ferritin reached a plateau in the middle tertile of capsule intake for all micronutrient supplementation types. A plateau was confirmed by statistical tests because women in the middle and highest tertiles of capsule intake had higher mean ferritin concentrations than did those in the lowest tertile (**Table 5**). However, there were no significant differences between the middle and highest tertiles of capsule intake within any of the micronutrient supplementation types (*P* = 0.20, 0.81, and 0.22 for the Fe30F, Fe60F, and MMN groups, respectively; results not shown).

**TABLE 5:** Plasma ferritin concentrations at week 30 of pregnancy by tertile of capsule intake and micronutrient supplementation type in a selected sample of rural Bangladeshi women participating in the MINIMat trial^1^

Micronutrient type	Lowest tertile (6–65 capsules)	Middle tertile (66–99 capsules)	Highest tertile (100–157 capsules)	*P*-interaction
Fe30F				
* n*	61	55	67	
Mean,^2^ μg/L	11.6 (9.58, 14.0)	18.9 (15.5, 23.1)	16.1 (14.0, 18.4)	
Unadjusted mean difference^3^	Reference	0.49 (0.24, 0.74)**	0.33 (0.09, 0.56)**	
Adjusted mean difference^4^	Reference	0.40 (0.17, 0.62)**	0.25 (0.03, 0.48)*	
Unadjusted response per capsule^5^	−0.005 (−0.016, 0.006)			
Adjusted response per capsule^4^	−0.001 (−0.012, 0.011)			
Fe60F				
* n*	76	66	61	
Mean,^2^ μg/L	11.5 (9.39, 14.0)	20.7 (17.1, 25.0)	20.7 (17.8, 24.1)	
Unadjusted mean difference^3^	Reference	0.59 (0.34, 0.85)**	0.59 (0.33, 0.85)**	
Adjusted mean difference^4^	Reference	0.52 (0.29, 0.75)**	0.55 (0.31, 0.78)**	
Unadjusted response per capsule^5^	0.013 (0.002, 0.025)			
Adjusted response per capsule^4^	0.011 (0.000, 0.022)			
MMN				
* n*	52	70	68	
Mean,^2^ μg/L	13.1 (10.6, 16.1)	16.8 (14.6, 19.3)	18.5 (16.0, 21.3)	
Unadjusted mean difference^3^	Reference	0.25 (0.02, 0.48)*	0.35 (0.12, 0.58)**	
Adjusted mean difference^4^	Reference	0.20 (0.00, 0.39)**	0.31 (0.11, 0.52)*	
Unadjusted response per capsule^5^	0.016 (0.003, 0.028)			
Adjusted response per capsule^4^	0.010 (−0.002, 0.021)			
Fe30F vs. Fe60F^6^				
Unadjusted response per capsule	−0.019 (−0.035, −0.004)			0.02
Adjusted response per capsule^4^	−0.013 (−0.028, 0.002)			0.08
MMN vs. Fe60F				
Unadjusted response per capsule	0.001 (−0.015, 0.016)			0.92
Adjusted response per capsule^4^	−0.001 (−0.016, 0.014)			0.89
Fe30F vs. MMN				
Unadjusted response per capsule	−0.020 (−0.035, −0.004)			0.01
Adjusted response per capsule^4^	−0.013 (−0.028, 0.003)			0.10

1 Data were analyzed by using general linear models.*,**Different from the lowest tertile: *P < 0.05, **P < 0.01. Fe30F, 30 mg Fe and 400 μg folic acid; Fe60F, 60 mg Fe and 400 μg folic acid; MINIMat, Maternal and Infant Nutrition Intervention, Matlab; MMN, multiple micronutrient supplement.

2 Values are geometric means (95% CIs).

3 Values are log_e_ mean differences (95% CIs).

4 Models adjusted for maternal formal years of education (none, 1–5 y, or ≥6 y), socioeconomic status (quintiles), food supplementation, week 14 micronutrient concentration (log_e_), and duration of supplementation in the analyses.

5 Response per capsule was evaluated within the first tertile of capsule intake; values are log_e_ transformed.

6 Comparison was made within the first tertile of capsule intake; values are log_e_ transformed.

To compare efficacy in dose-response per capsule between the micronutrient supplementation types, the first tertile of capsule intake (where a dose-response was observed) was selected. In adjusted linear regression analyses, ferritin was modeled as a function of the number of capsules taken, micronutrient supplementation type, and interaction between number of capsules and micronutrient supplementation type. Women in the Fe30F group tended to have a lower response per capsule than did women in the Fe60F group (β_adj_: −0.013; 95% CI: −0.028, 0.002; *P*-interaction = 0.08) and the MMN group (β_adj_: −0.013; 95% CI: −0.028, 0.003; *P*-interaction = 0.10) (Table 5). There was no significant difference in dose-response between women in the Fe60F and MMN groups (*P*-interaction = 0.89).

#### Zinc and dose-response.

Despite patterns that appeared in the Lowess plot (Figure 2D), with the use of regression analyses no dose-response relation between the total number of capsules taken and plasma zinc concentration was observed for any of the micronutrient supplementation types. Furthermore, there were no significant differences in zinc concentration between the tertiles of capsule intake for any of the micronutrient supplementation types (**Table 6**).

**TABLE 6:** Plasma zinc concentrations at week 30 of pregnancy by tertile of capsule intake and micronutrient supplementation type in a selected sample of rural Bangladeshi women participating in the MINIMat trial^1^

Micronutrient type	Lowest tertile (6–65 capsules)	Middle tertile (66–99 capsules)	Highest tertile (100–157 capsules)
Fe30F			
* n*	63	55	68
Mean,^2^ μmol/L	8.00 (7.39, 8.58)	7.24 (6.68, 7.84)	7.46 (6.89, 8.00)
Unadjusted mean difference^3^	Reference	−0.10 (−0.21, 0.02)	−0.07 (−0.18, 0.04)
Adjusted mean difference^4^	Reference	−0.08 (−0.20, 0.04)	−0.07 (−0.19, 0.04)
Fe60F			
* n*	69	66	62
Mean,^2^ μmol/L	7.61 (6.96, 8.25)	7.61 (7.03, 8.33)	7.54 (6.96, 8.25)
Unadjusted mean difference^3^	Reference	0.00 (−0.12, 0.12)	−0.00 (−0.12, 0.12)
Adjusted mean difference^4^	Reference	0.02 (−0.10, 0.14)	−0.00 (−0.12, 0.12)
MMN			
* n*	53	70	68
Mean,^2^ μmol/L	7.69 (7.10, 8.25)	7.77 (7.24, 8.25)	7.92 (7.54, 8.41)
Unadjusted mean difference^3^	Reference	0.01 (−0.09, 0.10)	0.03 (−0.07, 0.13)
Adjusted mean difference^4^	Reference	−0.00 (−0.10, 0.10)	0.03 (−0.08, 0.13)

1 Data were analyzed by using general linear models. Fe30F, 30 mg Fe and 400 μg folic acid; Fe60F, 60 mg Fe and 400 μg folic acid; MINIMat, Maternal and Infant Nutrition Intervention, Matlab; MMN, multiple micronutrient supplement.

2 Values are geometric means (95% CIs).

3 Values are log_e_ mean differences (95% CIs).

4 Models adjusted for maternal formal years of education (none, 1–5 y, or ≥6 y), socioeconomic status (quintiles), food supplementation, week 14 micronutrient concentration (log_e_), and duration of supplementation.

## Discussion

### 

In this study, women who were assigned to the MMN regimen had higher concentrations of plasma vitamin B-12 at week 30 of pregnancy, but no other differences between food and micronutrient regimens were observed. A per-capsule dose-response was observed for plasma folate and ferritin concentrations for all micronutrient supplementation types. The association between the number of capsules taken and plasma folate was linear, and efficacy in increasing folate concentration was comparable among micronutrient supplementation types. Plasma ferritin appeared to reach a plateau within the middle tertile of capsule intake for all 3 types, resulting in equivalent effectiveness of the 3 micronutrient supplementation regimens. In the first tertile of capsule intake, a similar dose-response relation was observed for the MMN and Fe60F supplementation types, whereas Fe30F had lower efficacy per capsule in increasing ferritin concentrations.

#### Vitamin B-12.

At week 30 of pregnancy, plasma vitamin B-12 concentrations were significantly higher among the women in the MMN group. However, there was no dose-response relation between the number of MMN capsules taken and plasma vitamin B-12 that could explain the higher plasma concentrations of vitamin B-12 at week 30 in the MMN group. This apparent contradiction in results may be explained by a pronounced response to a few capsules ingested, resulting in a plateau of response at a low capsule intake.

A positive effect of MMN supplementation on maternal plasma vitamin B-12 has also been observed among pregnant women in Nepal (17). Despite supplementation, plasma vitamin B-12 decreased from early to late gestation for all 3 micronutrient types in our study. Hemodilution and placental transport of vitamin B-12 to the fetus (18) may be possible explanations for the observed decrease in plasma concentrations of vitamin B-12.

#### Folate.

Plasma folate concentration at week 30 was comparable among all micronutrient supplementation regimens. We found a dose-response relation between the number of capsules taken and plasma folate for all supplementation types, ruling out the potential explanation that the lack of difference between regimens at week 30 was due to a lack of response in any of them. We did not find any significant differences in dose-response between the supplementation types, indicating that the effect on plasma folate was not modified by the dose of iron or the presence of other micronutrients.

In line with our findings, a meta-analysis that assessed the effect of folate intake on markers of folate status reported a dose-response relation between folate intake and concentrations of circulating folate among women of childbearing age (19). A positive effect of folate-containing regimens on maternal circulating folate has also been reported in previous studies (17, 20). Most of the women in our study (82%) had adequate plasma folate concentrations at week 14 (14), which was further increased by supplementation. There is some evidence that high maternal folate status in combination with low vitamin B-12 status may increase the risk of obesity and insulin resistance in the offspring (21). Approximately 35% of women in our study had vitamin B-12 deficiency in combination with normal plasma folate at week 14 (14), which increased to 64% at week 30. Children of these women may be more vulnerable to chronic metabolic diseases later in life. Further follow-up of this cohort will be helpful to evaluate the effect of high folate and low vitamin B-12 status of mothers on their offspring.

#### Ferritin.

There were no significant differences in plasma ferritin at week 30 of pregnancy between the micronutrient supplementation regimens. Although a dose-response in ferritin concentration per capsule was observed for all 3 micronutrient supplementation types, the response per capsule was lower among women in the Fe30F group. Despite this lower efficacy per capsule, the effectiveness of the regimens was equivalent among supplementation regimens. This may be explained by plasma ferritin reaching a plateau after an intake of ∼70 capsules for women in the Fe60F and MMN groups, although it continued to respond to higher capsule intake in the Fe30F group.

It has been suggested that, if started relatively early in pregnancy, supplementation with a 30-mg Fe regimen would be as effective as 60 mg Fe in increasing circulating ferritin in pregnant women (22). The important difference between the Fe30 and Fe60 regimens would be that Fe60 provides a larger amount of iron earlier in pregnancy and thus potentially an earlier timing of a higher ferritin concentration. The benefits of Fe60 in potentially providing an earlier peak in ferritin needs to be evaluated in relation to potential adverse effects of providing higher doses of iron in early pregnancy. The safety of prenatal iron supplementation has been discussed, and of concern is that elevated body iron stores have been associated with gestational diabetes mellitus and preeclampsia (23, 24) as well as the risk of cardiovascular diseases (25).

The effect of regimen as well as the increment per capsule on ferritin concentration was comparable between the Fe60F and MMN groups. The presence of other micronutrients in MMNs such as vitamins C and A, which can improve the absorption of iron (26), may explain this equivalence despite the lower content of iron in the MMN.

Despite a positive dose-response, maternal plasma ferritin was lower at week 30 of pregnancy than at week 14 for all 3 supplement types. A decrease in ferritin concentration regardless of supplementation with iron has been observed in other studies (27–29). Plasma volume expansion and subsequent hemodilution may explain the lower ferritin concentrations in late pregnancy.

#### Zinc.

The plasma zinc concentration was not responsive to supplementation in our study sample. The lack of response to prenatal supplementation regimens containing 15 mg Zn has been reported in a study conducted in Indonesia (30), whereas supplementation with MMNs containing 30 mg Zn increased plasma zinc concentrations by 0.5 μmol/L in a study in pregnant women in Nepal (17). The lower content of zinc (15 mg) compared with the study in Nepal (30 mg) and an inhibitory effect of iron on zinc absorption (31) may have compromised the amount of zinc absorbed in our sample. However, a study in Peru that compared serum zinc in pregnant women who used iron supplements with or without 15 mg Zn did find an effect on serum zinc (32). A difference between our study and the Peruvian study is that the women in our study appeared to have a lower baseline zinc status.

The strength of this study is the randomized design in combination with careful assessment of adherence, enabling both an evaluation of the effectiveness of the supplementation regimens as well as of the efficacy per capsule of the supplement. However, there are some limitations that need to be acknowledged. Because C-reactive protein (CRP) was elevated in only 4% of the women at week 14 of pregnancy (14), and in 7% of a subset (*n* = 95) of women at week 30, we decided not to measure CRP in all women at week 30; thus, the possibility of overestimating plasma ferritin and/or underestimating plasma zinc at week 30 of pregnancy due to the presence of infection cannot be excluded. Furthermore, although the use of eDEM has been considered to be one of the most accurate methods for measuring adherence, the device cannot confirm that the capsule actually was ingested. However, observed dose-response relations between the number of capsules taken and folate and ferritin concentrations suggest that this was not a problem. On a few occasions, the pill bottles were opened >3 times/d, which suggests a risk for overestimation of capsule intake; however, that would not have been possible because the pill bottles were refilled with 35 capsules/mo. Furthermore, to minimize this problem, we recorded >3 daily bottle openings as 3 openings. Finally, we did not take blood samples later than 30 wk of gestation, because it is customary for some women experiencing their first pregnancy to leave their houses to give birth in their maternal homes, which created difficulties for follow-up visits. Although measuring early effects of supplementation may be of greater relevance for fetal development, it would have been of interest to get a later assessment of maternal micronutrient status toward the end of pregnancy.

In conclusion, women assigned to the MMN regimen had a higher concentration of plasma vitamin B-12, although the public health significance of the size of the effect is uncertain. Despite the lower dose of iron in the MMNs and Fe30 supplements, these regimens produced effects similar to those of Fe60 on women’s ferritin in late pregnancy. The potential health benefits or risks of providing a larger amount of iron by Fe60 in early pregnancy need to be evaluated further.

## Footnotes

6 Abbreviations used: CRP, C-reactive protein; Fe30F, 30 mg Fe and 400 μg folic acid; Fe60F, 60 mg Fe and 400 μg folic acid; MINIMat, Maternal and Infant Nutrition Intervention, Matlab; MMN, multiple micronutrient supplement; β_adj_, adjusted β coefficient.
